# Analysis of exome data in a UK cohort of 603 patients with syndromic orofacial clefting identifies causal molecular pathways

**DOI:** 10.1093/hmg/ddad023

**Published:** 2023-03-27

**Authors:** Kate Wilson, Dianne F Newbury, Usha Kini

**Affiliations:** Oxford Centre for Genomic Medicine, Oxford University Hospitals NHS Foundation Trust, Oxford OX3 7HE, UK; Department of Biological and Medical Sciences, Faculty of Health and Life Sciences, Oxford Brookes University, Oxford OX3 0BP, UK; Centre for Functional Genomics, Oxford Brookes University, Oxford OX3 0BP, UK; Oxford Centre for Genomic Medicine, Oxford University Hospitals NHS Foundation Trust, Oxford OX3 7HE, UK; Spires Cleft Centre, John Radcliffe Hospital, Oxford OX3 9DU, UK; Radcliffe Department of Medicine, University of Oxford OX3 9DU, UK

## Abstract

Orofacial cleft (OC) is a common congenital anomaly in humans, which has lifelong implications for affected individuals. This disorder can be classified as syndromic or non-syndromic depending on the presence or absence of additional physical or neurodevelopmental abnormalities, respectively. Non-syndromic cleft is often non-familial in nature and has a complex aetiology, whereas syndromic forms tend to be monogenic. Although individual OC-related syndromes have been frequently described in the medical literature, there has not been a comprehensive review *across* syndromes, thereby leaving a gap in our knowledge, which this paper aims to address. Six hundred and three patients with cleft-related human phenotype ontology terms were identified within the Deciphering Developmental Disorders study. Genes carrying pathogenic/likely pathogenic variants were identified and reviewed enabling a diagnostic yield of 36.5%. In total, 124 candidate genes for syndromic OC were identified, including 34 new genes that should be considered for inclusion in clinical clefting panels. Functional enrichment and gene expression analyses identified three key processes that were significantly overrepresented in syndromic OC gene lists: embryonic morphogenesis, protein stability and chromatin organization. Comparison with non-syndromic OC gene networks led us to propose that chromatin remodelling specifically contributes to the aetiology of syndromic OC. Disease-driven gene discovery is a valid approach to gene identification and curation of gene panels. Through this approach, we have started to unravel common molecular pathways contributing to syndromic orofacial clefting.

## Introduction

Orofacial cleft (OC) is a common congenital anomaly in humans, which has lifelong implications for affected individuals. The incidence of OC in England, Wales and Northern Ireland is 15 per 10 000 live births (1). Cleft lip (CL) and cleft lip and palate (CLP) have historically been considered variants of the same defect with differing severity as they share a common anomaly of the primary palate (2). Cleft palate only (CPO) is associated with a defect of the secondary palate and is thus considered separate to CL and CLP due to the distinct developmental origins of the primary and secondary palates, although shared aetiological factors are still described. Subclinical phenotypes of cleft palate include submucous cleft palate and bifid uvula (2).

Syndromic OC is diagnosed when cleft is associated with additional physical or neurodevelopmental abnormalities (2). Syndromic OC accounts for ~ 30% of OC and is usually monogenic in nature, although variable expressivity and incomplete penetrance are widespread (3,4). The Genomics England PanelApp (PA) (https://panelapp.genomicsengland.co.uk) is a reputable, publicly available database of regularly updated and curated virtual gene panels that is a valuable reference tool for genes associated with syndromic OC (5). At the time of writing, the PA Clefting panel contained 142 genes for use in a clinical setting, suggesting that OC is a feature of numerous syndromes. Although individual OC-related syndromes have been frequently described in the medical literature, they are usually classified according to co-occurring features. There has not been a comprehensive review *across* OC syndromes, thereby leaving a gap in our knowledge.

In contrast to syndromic OC, the majority of cases of non-syndromic OC are non-familial and occur as an isolated congenital anomaly with a multifactorial aetiology involving both genetic and environmental factors, particularly for cleft lip +/− palate (CL/P) (6,7). Accordingly, the majority of gene mapping studies in non-syndromic OC have applied association and linkage methods. More than 40 loci have been identified to date and together these account for > 25% of risk (8). Replicated risk loci include *IRF6, GRHL3, PAX7, DCAF4L2, MAFB, NOG, NTN1* and *WTN5A* (4,7). More recently, exome sequencing studies have identified non-syndromic OC families with a Mendelian single gene aetiology including pathogenic variants in *CDH1, CTNND1, PLEKHA7, PLEKHA5, FGFR, ARHGAP29* and *DLG1* (9–11) but these seem to be the exception rather than the rule. Network analyses of genes associated with non-syndromic OC show a consistent overrepresentation of broad developmental gene ontology (GO) terms such as ‘embryonic development’ and, in particular, the WNT signalling pathways, although these are not ubiquitously described (12,13). Meta-analyses suggest that variants associated with non-syndromic OC fall within open chromatin regions that form enhancers during early embryonic development and/or loci that map to developmental transcription factors (6,13,14). These findings suggest that regulatory cascades during early embryonic development are critical to non-syndromic OC.

In general, genetic factors identified in both syndromic and non-syndromic forms of OC do not neatly map onto clinical OC subtypes (CPO, CL/P) or associated phenotypes. Instead, it seems likely that both rare and common variants contribute across all OCs and that some genes contribute across multiple clinical subgroupings (e.g. *CDH1*, *CTNND1*, *FGFR1*, *GRHL3* and *SATB2)* (4,15). The consideration of genes that contribute between and across subtypes will be crucial to our understanding of this heterogeneity.

Genetic testing through exome and genome sequencing has become increasingly available in clinical and research settings creating an opportunity to enhance our knowledge of the diagnoses, molecular pathways and networks associated with syndromic OC. The Deciphering Developmental Disorders (DDD) is a national collaborative project in the United Kingdom established to facilitate the translation of genomic sequencing technologies into the National Health Service. It includes over 13 000 patients with congenital anomalies and/or undiagnosed developmental disorders, all of whom have broad clinical phenotypes and available exome data (16,17). Patients with isolated, single congenital anomalies do not meet the inclusion criteria for DDD and hence patients with non-syndromic OC would be excluded from this cohort. By reviewing the variants identified in patients with syndromic OC, through exome and array-CGH data within the DDD study, this paper aims to understand the spectrum of rare disorders associated with syndromic OC, to enhance our understanding of the origin and causes of clefting and enable expansion of curated clefting gene panels used in a clinical setting. We aim to further probe the molecular pathways associated with syndromic OC and study the differences between syndromic and non-syndromic OC. This investigation represents the first step in understanding causes of clefting within and between syndromes and provides an initial overview of similarities and differences between genetic drivers of OC in syndromic and non-syndromic forms.

## Results

### Patient cohort

Approximately 5% of the patients with data available through the DDD study, which includes individuals with undiagnosed developmental syndromes (DataFreeze 2017-12-15: 13 612 probands), had the human phenotype ontology (HPO) terms ‘cleft’ and/or ‘bifid uvula’ (631/13 612, 4.6%) (Supplementary Material, Table S1) (16). After excluding individuals with non-orofacial clefting (28/631, 4.4%), 603 patients remained [hereafter referred to as the OxSOC (Oxford Syndromic OC) cohort] including 345 males (57.2%) and 258 females (42.8%), aged 0–44.55 (median 4.50 years). Seventy-four percent of patients presented with CPO (448 of 603, 74.3%), 21.9% (132 of 603) with CL/P and 3.8% (23 of 603) with unspecified oral cleft (i.e. cleft phenotype not specific by clinician) (Supplementary Material, Table S1). A male predominance was observed in both clinical subsets (1.21 M:1F for CPO, 2.14 M:1F for CL/P). This excess was statistically significant when compared with the UK population [0.96 M:1F, 2011 census—χ^2^ = 15.75(1), *P* = 7.2 ×10^−5^] but not compared with the DDD study from which the samples were drawn [1.38 M:1F—χ^2^ = 0.22(1), *P* = 0.64]. The male excess was observed across cases with and without ID.

Excluding OC-related HPO terms, 1277 different HPO terms were assigned 4731 times across the 603 OC patients. Affecting 9.84 of the HPO phenotypic systems. The most frequently affected systems were abnormalities of the head or neck (79% of patients, 72% of CL/P cases, 82% of CPO cases) (in particular facial dysmorphology), abnormalities of the musculoskeletal system (77% of patients, 67% of CL/P cases, 81% of CPO cases) (in particular the facial skeleton) and abnormalities of the nervous system (75% of patients, 64% of CL/P cases, 79% of CPO cases) (in particular microcephaly and seizures). These three systems were more commonly affected in patients with syndromic CPO [χ^2^ = 5.915(1), *P* = 0.015, χ^2^ = 11.717(1), *P* = 0.0006, χ^2^ = 11.032(1), *P* = 0.0009, respectively] (Supplementary Material, Table S2). Abnormalities of limbs, eyes, ears, integument, cardiovascular system, digestive system and growth were also commonly reported and abnormalities of growth were, again, more common amongst cases with syndromic CPO [23% of patients, 14% of CL/P cases, 25% of CPO cases, χ^2^ = 7.317(1), *P* = 0.0068] (Supplementary Material, Table S2). The second most common phenotypic feature (after orofacial clefting) in the OC-selected cohort was intellectual disability (ID); 43% of patients (259/603) had moderate to profound ID and a further 8.5% (51/603) had mild or borderline ID. The frequency of common HPO terms did not differ between patients with or without ID (Supplementary Material, Table S3). The most commonly described features (after orofacial clefting and ID) were hypertelorism (15.6% of patients), micrognathia (13.1% of patients), delayed speech and language (12.9% of patients), microcephaly (10.8% of patients) and ventricular septal defects (7.3% of patients) (Supplementary Material, Table S3).

Consanguinity was recorded in 4.7% of OC cases (Supplementary Material, Table S2), as compared with 10.4% in the general UK population (18). Twenty-seven mothers (4.49%) experienced diabetes during pregnancy and four children (0.66%) were exposed to anti-epileptic drugs during pregnancy (Supplementary Material, Table S2). Individuals with these risk factors are noted in Supplementary Material, Tables S2, S4 and S5, but were not excluded from analyses as they do not preclude a genetic aetiology.

Through examination of exome sequence and array-CGH data, DatabasE of genomiC varIation and Phenotype in Humans using Ensembl Resources (DECIPHER) listed 836 ‘plausibly diagnostic’ sequence variants in 413 of the 603 (68.5%) patients On average, each patient had 7.85 non-OC HPO terms (median = 7, mode = 5, range = 0–27) and ‘plausibly diagnostic’ copy number variants (CNVs) were recorded in 45 cases (7.5%), 20 of whom also had ‘plausibly diagnostic’ sequence variants. Each of these variants were assessed according to standard American College of Medical Genetics (ACMG) and Association for Molecular Pathology (AMP) guidelines (19) identifying 238 Pathogenic/Likely Pathogenic (P/LP) variants [214 single nucleotide variants (SNVs) (Supplementary Material, Table S4), 24 CNVs (Supplementary Material, Table S5)] in 220 patients, giving a diagnostic yield of 36.5%. Yields were similar across diagnostic classes; 33.3% in patients with CL/P (SNVs in 38 patients, CNVs in 6 patients), 37.1% in patients with CPO (SNVs in 150 patients, CNVs in 16 patients) and 43.5% in patients with unspecified cleft (HPO term: oral cleft, SNVs in 9 patients, a CNV in 1 patient) (Supplementary Material, Tables S4 and S5). Overall, ~4% (23/603) of patients had a P/LP CNV that partially or fully explained their phenotype (Supplementary Material, Table S5). Six patients had compound genotypes; one with two CNVs, and five with compound SNVs (Supplementary Material, Tables S4 and S5).

Eighty percent (192/238) of P/LP variants were present in a heterozygous form, consistent with a dominant pattern of inheritance. Twelve variants (5.0%) were identified in a homozygous state and 12 compound heterozygotes were present, consistent with a recessive pattern of inheritance. Inheritance information was available for 86.1% (205/238) of P/LP variants and 72.7% (149/205) of these variants were *de novo*. The parental phenotype data did not indicate the presence of a cleft in these cases. Twenty variants were inherited in a heterozygous form (4 paternal, 16 maternal), 20 in a biallelic form (12 compound heterozygous and 8 biparental) and 4 were hemizygous variants. All variants and inheritance are shown in Supplementary Material, Tables S4 and S5.

One hundred and twenty-four genes with P/LP variants were identified in the OxSOC cohort (Supplementary Material, Table S6). Twenty genes had P/LP events in three or more unrelated patients (Supplementary Material, Table S6). *SATB2* was the most commonly identified gene and harboured potentially pathogenic variants in 2.7% (16/603) of the total patients investigated, all of whom had CPO (Supplementary Material, Table S6). *KMT2D* variants accounted for a further 1.0% (6/603) of patients (Supplementary Material, Table S6). Interestingly, all of the *KMT2D* patients also presented with CPO, although numbers in this group were small. All other genes each accounted for < 1% of the cohort (Table 1 and Supplementary Material, Table S6).

**Table 1:** Genes with three or more P/LP variants in OxSOC cohort

Gene	# cases in OxSOC	Clefting type in OxSOC	Clefting type in literature	Included in PA Clefting panel?	ID/GDD in OxSOC? (Y/N)	ID/GDD in literature? (Y/N)
*SATB2*	16	CPO	CPO	Yes, green	Y	Y
*KMT2D*	6	CPO	CL/P	Yes, green	Y	Y
*PGAP3*	5	CPO	CPO	No	Y	Y
*CHD7*	5	2 CL/P, 2 CP, 1 CP + OC	CL/P	Yes, green	Y	Y
*EFTUD2*	5	CPO	CPO	Yes, green	Y	Y
*ANKRD11*	4	3 CPO, 1 CL/P	CPO	Yes, green	Y	Y
*CTCF*	4	2 CPO, 2 CL/P	CL/P, CPO	Yes, green	Y	Y
*CTNND1*	4	3 CL/P, 1 CPO	CL/P	Yes, green	Y	N
*PIEZO2*	4	CPO	CPO	Yes, green	Y	Y
*ADNP*	3	1 CL/P, 2 CPO	CPO	No	Y	Y
*ARID1B*	3	CPO	CPO	No	Y	Y
*COL2A1*	3	CPO	CPO	Yes, green	N	N
*GLI2*	3	1 OC, 2 CL/P	CL/P	No	Y	N
*HNRNPK*	3	1 OC, 2 CPO	CPO	No	Y	Y
*KAT6B*	3	CPO	CL/P	Yes, red	Y	Y
*MED13L*	3	CPO	CPO	Yes, amber	Y	Y
*MID1*	3	1 OC, 2 CL/P	CL/P	Yes, green	N	Y
*SMC1A*	3	CPO	CPO	Yes, green	Y	Y
*STAG2*	3	CPO	CL/P	No	Y	Y
*ZC4H2*	3	CPO	CPO	No	Y	Y

ID = intellectual disability; GDD = global developmental delay

For full OxSOC gene list, see Table S6

PA uses a traffic light system under which genes considered to be of clinical use are tagged as ‘green’. Green genes must be described in three or more unrelated cases in which the phenotype is observed, even if there are additional phenotypic features. Cases can be spread across independent studies in the literature. When combined with the literature, 82 genes carried P/LP variants in three or more unrelated cases with OC and so could be considered ‘green’ genes (5) (Supplementary Material, Tables S6 and S7). These genes are hereafter referred to as the OxSOC green genes (Supplementary Material, Table S8). Twenty-seven of the OxSOC green genes are not in the current PA Clefting panel (*ADNP*, *ARID1A*, *ARID1B*, *CHD3*, *CHD4*, *CNTNAP1*, *ECEL1*, *FGFR3*, *GLI2*, *HNRNPK, KMT2A, NEB, NOTCH2, PGAP3, POGZ, PUF60, RAD21, SETD2, SMARCA4, SMARCB1, STAG2, TBL1XR1, TCF12, TRAF7, TRRAP, UBE3B, ZC4H2)* and seven further genes (*B4GALT7, DDX3X, FBXO11, KAT6B, MED12, MED13L, PGM1*) are currently classified as red or amber, thereby increasing the number of green genes in the panel by 34 (23.9%, Supplementary Material, Table S7). Twenty six of these 34 genes (76.5%) carried P/LP variants in patients with CPO, two (5.9%) had P/LP variants in patients with CL/P and six (17.6%) had variants across multiple patients some of whom presented with CL/P and others with CPO.

### Network analyses

**Figure 1:**
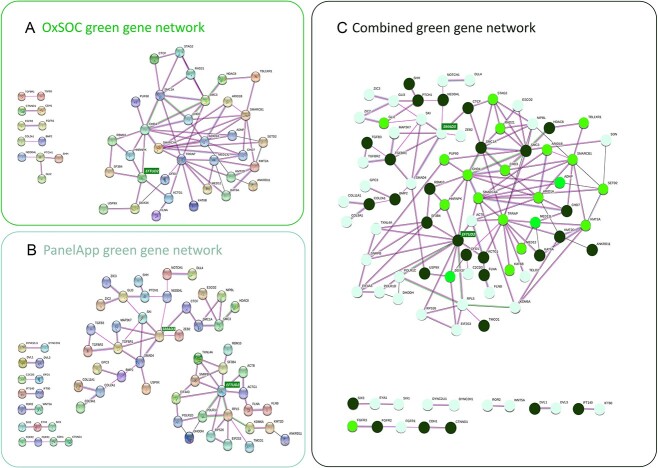
Cleft Gene Networks. Genes are represented by balls and relationships by lines. Colour of the line depicts the type of evidence for the relationship; pink lines represent experimental evidence, green lines represent shared gene neighbourhood, black lines represent co-expression, blue lines represent protein homology and red lines represent gene fusions. The genes added by the OxSOC network (1a) allowed joined two distinct networks seen in the PA Clefting gene set (1b) through the connection of the EFTUD2 and SMAD3 hubs (1c). These hub genes are highlighted in green boxes. (**A**) Network of interconnected genes identified in the OxSOC green genes. Thirty-four of the 82 OxSOC green genes form a network connected by 66 edges with an average node degree of 3.88 (PPI enrichment, *P* = 1 × 10^−16^). (**B**) Network of existing PA green cleft genes. Forty-eight of the 142 PA green genes form two distinct networks connected by 62 edges with an average node degree of 2.62 (PPI enrichment, *P* = 7.22 × 10^−11^). (**C**) Combined cleft network (OxSOC and PA green genes). Seventy-four of the 176 green genes form a network connected by 141 edges with an average node degree of 3.81 (PPI enrichment, *P* < 1 × 10^−16^). Genes that are found only in PA green list are shown as pastel green circles, genes that are included in both PA and OxSOC green lists are shown as dark green circles, genes that are found only in OxSOC green list are shown as bright green circles.

STRING analyses of the 82 OxSOC green genes revealed a highly connected network (Protein-protein interaction (PPI) enrichment, *P* = 1 × 10^−16^), which included 34 genes (41%) connected by 66 edges (average node degree of 3.88), in addition to 5 smaller connections with 2–4 nodes (Fig. 1A, Supplementary Material, Table S8). The most connected genes in the identified network were *CHD4* and *TRRAP* (each with 10 edges) (Supplementary Material, Tables S8 and S9). Ten of the 11 genes with five or more connections (*ARID1A*, *CHD3*, *CHD4*, *KMT2A*, *MED13L*, *SMARCA4*, *SMARCB1*, *SMC1A*, *SMC3* and *TRRAP*) functioned in chromatin remodelling, a process that is essential to cellular differentiation and maintenance and has been implicated across a range of neurological disorders (20,21). The majority of these patients (15 of 17 patients with variants in 8 of 10 genes) presented with CPO (Supplementary Material, Table S4). Of note, 41% of the probands carrying P/LP variants in these 10 genes did not present with ID.

Seventeen Gene Ontology (GO) terms (3 GO components and 14 GO processes) were overrepresented (False Discovery Rate (FDR) < 0.0001 and ≥ 1.2-fold) in the OxSOC green gene list (Supplementary Material, Table S10). These terms centred around embryonic morphogenesis and chromatin regulation (Supplementary Material, Table S10) and included 34 genes that carried P/LP variants in 74 OxSOC patients, 54 (73.0%) of whom presented with CPO and 17 (23.0%) of whom presented with CL/P. The most overrepresented terms were cohesin complexes (GO:0000118, 1.98× enrichment, FDR = 3.2 × 10^−5^) and limb bud formation (GO:0060174, 1.94× enrichment, FDR = 4.68 × 10^−5^).

The OxSOC green gene list (*N* = 82) was combined with the green genes in the existing PA Clefting panel (*N* = 142) to generate an updated list of 176 green cleft genes (hereafter referred to as the combined green gene list; Supplementary Material, Table S8). Forty-eight genes were common to both the OxSOC green gene list and the PA Clefting panel. Network analysis of this extended list showed that the OxSOC green genes joined two distinct networks in the existing PA Clefting panel (Fig. 1B) through the connection of the *EFTUD2* and *SMAD3* hubs (Fig. 1C, green highlights). The merged network included 74 genes connected by 141 edges (average node degree of 3.81, PPI enrichment, *P* < 1 × 10^−16^) (Supplementary Material, Table S11). Eighty-six OxSOC patients carried P/LP variants in these 74 genes, 67 (77.9%) with CPO, 15 (17.4%) with CL/P and 4 (4.7%) with unspecified OC. Twenty-three genes had five or more nodes; 11 of these genes (*ARID1A*, *CHD3*, *CHD4*, *KMT2A*, *MED13L*, *RAD21*, *SETD2*, *SMARCA4*, *SMARCB1*, *STAG2* and *TRRAP*) were not included in the PA Clefting panel as green genes (Fig. 1C, Supplementary Material, Table S8). Again, patients carrying P/LP variants in these genes were more likely to present with CPO [32 of 34 patients (94.1%) across 23 genes with 5 or more nodes and 17/18 patients (94.4%) across 11 new genes].

Enrichment analyses of the combined green gene list (*N* = 176) revealed a significant overrepresentation across 54 GO terms (1 GO component and 53 GO processes) with FDR < 0.0001 and ≥ 1.2-fold enrichment (Supplementary Material, Table S12). The most significantly enriched GO terms again centred around morphogenesis and differentiation (minFDR = 2 × 10^−24^, GO:0060173) and included chromatid cohesion (GO:0007062; FDR = 1.01 × 10^−6^, 1.33-fold enrichment) and neuronal cell fate (GO:0048663, FDR = 1.29 × 10^−7^, 1.23-fold enrichment). Of these three GO categories, GO:0060173 was primarily associated with CL/P in OxSOC patients [of 19 patients with OC type classified, 11 (57.9%) had CL/P, *z* = 3.535, *P* = 0.0004]. In contrast, genes in GO:0007062 and GO:0048663 categories were found to carry P/LP variants primarily in OxSOC patients with CPO (83.3% and 85.7% of classified patients, respectively) at a level consistent with presentation in the OxSOC cohort as a whole (77.2% of classified patients CPO).

**Figure 2:**
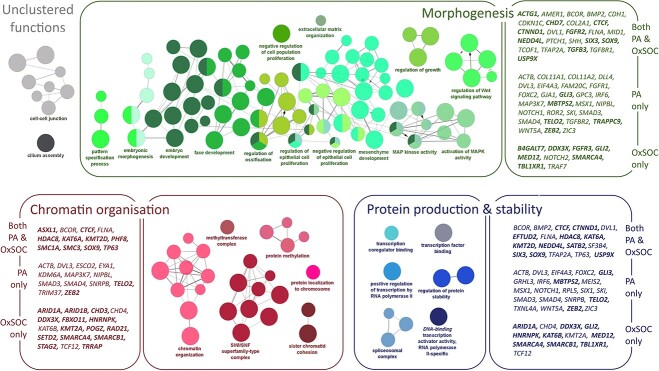
Functional network clusters in the Combined green gene list. Colour of node represents clustered GO functions, size of node represents significance of GO term. Functional clusters primarily centre around embryonic morphogenesis (shown in green), chromatin organization and remodelling (shown in red) and protein production and stability (shown in blue). Genes contributing to each function are shown in boxes to the side of the grouped functions. Gene names are split into three sections; the top section shows genes that were present in both the PA green gene list and the OxSOC green gene list, the middle section shows genes that were present in the PA green gene list but not the OxSOC green gene list, the bottom section shows genes that were present in the OxSOC green gene list but not the PA green gene list. Genes that were associated with moderate to severe forms of intellectual in OxSOC cases or in the literature or in HPO (https://hpo.jax.org/app/) under the terms ‘intellectual disability’ or ‘global developmental delay’ (moderate, severe or profound) are highlighted in bold.

ClueGO analysis allowed clustering of related gene functions across the combined green gene list and highlighted 28 functional groups with FDR < 0.5 (Supplementary Material, Table S13). The functional groupings formed three super-clusters; one around embryonic morphogenesis and cellular differentiation (14 functional groups, 61 genes, 107 GO-terms—green nodes in Fig. 2), another around chromatin organization and remodelling (6 functional groups, 42 genes, 27 GO-terms—red nodes in Fig. 2) and one around protein production and stability (6 functional groups, 52 genes, 9 GO-terms—blue nodes in Fig. 2). Two unclustered functional groups remained: cell–cell junction (15 genes, 8 GO-terms) and cilium assembly (10 genes, 1 GO-term) (Fig. 2, Supplementary Material, Table S13). Both the PA Clefting panel genes and OxSOC genes contributed to each of these super-clusters, as did genes known to be associated with ID (Fig. 2, Supplementary Material, Table S13). In line with the enrichment analysis above, genes in the embryonic morphogenesis super-cluster carried P/LP variants in a higher proportion of CL/P OxSOC patients than expected [18 of 47 (34.6%) patients with classified OC, presented with CL/P, *z* = 2.402, *P* = 0.016]. Genes included in the chromatin organization and protein production super-clusters and in the unclustered functional groups, all had a higher number of P/LP variants in CPO patients in OxSOC [43 of 55 classified patients (78.2%), 2 of 4 classified patients (50.0%) and 10 of 16 classified patients (62.5%), respectively]. These were at a level that reflected the CPO enrichment in OxSOC as a whole (77.2% of classified patients).

### Network comparison between clinical categories

To further investigate the functions of genes implicated across different types of OC, the combined green gene list was split according to clinical presentation to allow comparison between genes that cause syndromic CL/P and those that cause syndromic CPO. As the DDD cohort does not include non-syndromic cases, genes from the combined green gene list were compared with a separate list of genes reported to contribute to non-syndromic forms of OC. This list was downloaded from CleftGeneDB, a database of experimentally identified genes associated with CL and/or palate in humans (https://bioinfo.uth.edu/CleftGeneDB/). It is generally considered that non-syndromic forms of OC tend to be polygenic. As such, there is no gold-standard or complete gene list. We chose this list because it provides an overview of current literature in relation to non-syndromic genes and is manually curated by developmental biologists. From the combined green gene list, 53% of genes were associated with syndromic CPO and 44% with syndromic CL/P in the OxSOC patients and/or current literature (Table 1, Supplementary Material, Table S14). All but one of these genes (*ARHGAP29*) were associated with syndromic OC (Table 1, Supplementary Material, Table S14). After the addition of *ARHGAP29*, the non-syndromic gene list included 106 genes, 17 of which were also present in the combined green gene list (Supplementary Material, Table S14). These 17 genes were included in both syndromic and non-syndromic network analyses below. All non-syndromic genes were related to CL/P.

**Figure 3:**
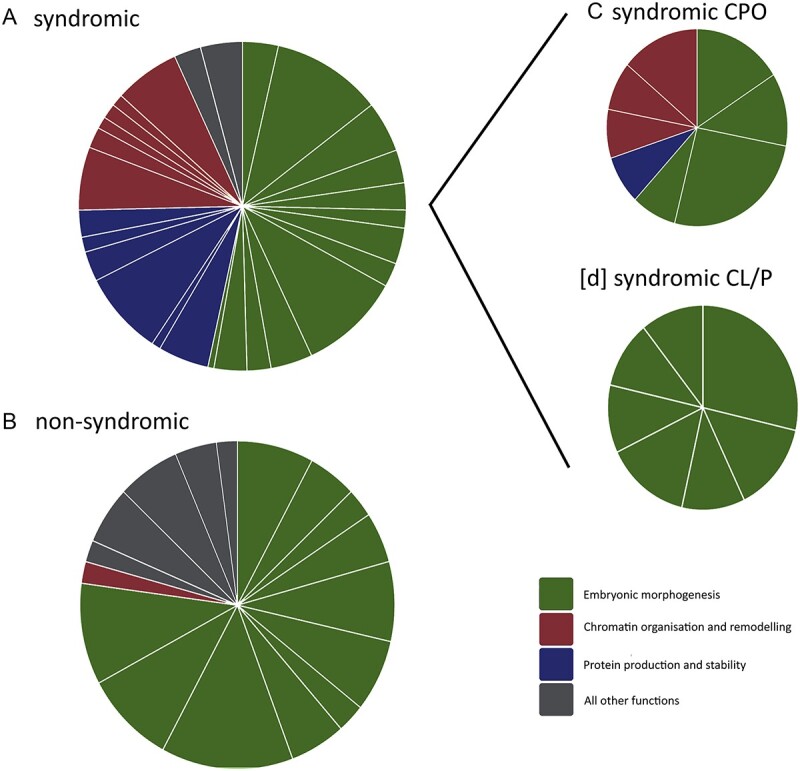
Functional network clusters in clinical subsets. Functional clusters were assigned within clueGo. Each slice of the pie shows a functional cluster. Size of slices represents the number of genes assigned to a given function. Colour of slices represents functional groups as identified in Figure 2; embryonic morphogenesis (shown in green), chromatin organization and remodelling (shown in red) and protein production and stability (shown in blue). All other functions are shown in grey. Full functional labels are listed in Supplementary Material, Table S15. Each quadrant shows a different gene set; (**A**) All OxSOC/PA combined green gene list (*N* = 176, primarily syndromic), (**B**) comparison CleftDB non-syndromic genes (*N* = 106, primarily CL/P), (**C**) OxSOC/PA green genes associated with CPO (*N* = 93), (**D**) OxSOC/PA green genes associated with Cleft Lip and/or Palate (CL/P, *N* = 77).

Network analyses and clustering of these clinical subgroups showed that the functional enrichment of chromatin organization and protein stability observed in the combined green gene list is driven by genes associated with syndromic CPO (Fig. 3C). In contrast, genes associated with syndromic CL/P showed enrichment only in embryonic morphogenesis-related functions (Fig. 3D, Supplementary Material, Table S15). Non-syndromic OC genes were also enriched in embryonic morphogenesis functions, cortical actin cytoskeleton, receptor ligand activity and alcohol and folate metabolism (Fig. 3B).

### Expression analysis

To further investigate the possibility that clinical subgroups map onto distinct functional networks, developmental gene expression patterns were examined between syndromic (*N* = 175) and non-syndromic (*N* = 106) gene lists, and syndromic CL/P (*N* = 77) and syndromic CPO (*N* = 93) gene lists in samples from human brain and dental pulp and from mouse mandibular and maxillary arches and palate tissue. Comparisons were also made across the three identified functional clusters [embryonic morphogenesis (*N* = 61), chromatin organization (*N* = 42) and protein stability (*N* = 52)].

**Figure 4:**
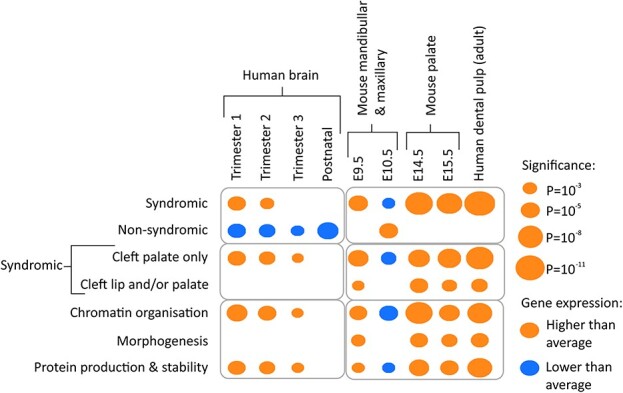
Expression of gene subsets in brain and palate tissues. Average expression levels of all genes within each clinical gene subset (syndromic, non-syndromic, CPO, Cleft Lip and/or Palate CL/P) and functional network (morphogenesis, chromatin organization and protein production) were compared against the average expression levels of all protein coding genes within the same tissues. Gene lists that show higher than average expression are denoted by orange dots, gene lists that show lower than average expression are denoted by blue dots. The size of the dot represents the level of significance.

Genes associated with syndromic OC showed a relatively higher level of expression across all palatal tissues investigated, from E9.5 to E15.5 in mice and in human dental pulp (Fig. 4). The only exception to this was at time E10.5 when a low level of relative expression was noted (Fig. 4). Interestingly, at this time-point, the non-syndromic OC gene set showed higher than average expression (Fig. 4). Palatal expression of syndromic genes was most pronounced at later time-points, when the palatal shelves fuse to form the palate (min adjusted-*P* = 9.57 × 10^−10^, syndromic genes in mouse palate, E14.5). Genes related to syndromic CPO showed a higher level of expression than syndromic CL/P (min CPO adjusted-*P* = 7.21 × 10^−10^, human dental pulp, min CL/P adjusted-*P* = 1.23 × 10^−4^, mouse palate, E14.5) as did the chromatin organization and protein stability gene clusters (min adjusted-*P* = 9.52 × 10^−10^, chromatin organization in mouse palate, E14.5) (Fig. 4).

In the brain, specific gene sets were observed to have characteristic expression patterns prenatally (Fig. 4). Syndromic genes were expressed at a level above average (min adjusted-*P* = 2.07 × 10^−4^, Trimester 1), whereas non-syndromic genes were expressed at a lower level than other protein-coding genes (min adjusted-*P* = 4.44 × 10^−6^, postnatal). The relative increased expression of the syndromic genes appeared to be driven by the CPO subset of genes (min adjusted-*P* = 1.91 × 10^−4^) with no contribution from the CL/P genes and specifically, came from the gene networks related to chromatin organization and protein stability (min adjusted-*P* = 9.66 × 10^−6^ and 1.05 × 10^−4^) (Fig. 4). All *P*-values can be found in Supplementary Material, Table S16. These patterns coincide with that observed in the GO analyses, in which CL/P patients were overrepresented in embryonic morphogenesis functional classes.

## Discussion

This report represents one of the largest genetic studies of syndromic OC to date involving 603 patients (the OxSOC cohort) and, importantly, is one of only a few studies focussing on monogenic causes of syndromic OC across syndromes. Our genomic analyses demonstrate the utility of exome sequencing and array-CGH as a diagnostic tool for these patients and add 34 genes to current diagnostic panels. Network analyses revealed systematic differences in the molecular mechanisms underlying not only syndromic and non-syndromic OC, but also syndromic CPO and syndromic CLP. Together, these findings provide a primary overview of the genetic architecture of syndromic OC allowing a comparison to that described in non-syndromic forms of cleft.

This cohort represents syndromic OC alone and hence unsurprisingly there was an excess of patients with CPO (74%) compared with CLP (22%). Contrary to the previously reported male to female ratios of 2:1 for CLP and 1:1.77 for CPO, which includes both syndromic and non-syndromic cleft types, our cohort showed an excess of males for both syndromic CLP and syndromic CPO (22). This appeared to coincide with the sex ratios for all patients recruited to the DDD study in general (16). In addition, an analysis of associated ID in these patients did not identify a correlation, thereby dismissing the possibility of the male excess in the cohort being explained by a link to X-linked ID. Consanguinity was reported in only 4.7% of this cohort (compared with 10.4% in the general UK population), which is merely a reflection of the fact that not all consanguinity results in cleft-related genetic disorders and that the majority of OC genes identified showed dominant inheritance.

Pathogenic or Likely Pathogenic variants were identified in 220 of the 603 probands (36.5%) investigated in this study, all of whom were selected on the presence of OC and had exome and CNV data available. The diagnostic yield was similar across all types of cleft investigated and in keeping with that reported in the literature (16,17). Nine genes (*ANKRD11*, *CHD7, CTCF*, *CTNND1, EFTUD2*, *KMT2D*, *PGAP3*, *PIEZO2* and *SATB2*) accounted for approximately one-quarter (51 of 214, 23.8%) of the patients with P/LP SNVs, including 42 of 167 patients with CPO (25.1%) and 8 of 38 patients with CL/P (21.1%) (Supplementary Material, Table S4). Six genes (*CHD7, CTCF, CTNND1, GLI2, TFAP2A* and *TP63*) carried P/LP variants across two or more OxSOC CL/P patients, whereas 37 genes carried P/LP variants across two or more OxSOC CPO patients. In particular, *SATB2, PGAP3, KMT2D, EFTUD2* and *PIEZO2*, all of which were reported in four or more OxSOC CPO cases. The most commonly reported gene across all OxSOC cases was *SATB2.* P/LP variants (both CNVs and SNVs) in this gene accounted for 2.65% (16/603) of patients investigated and 7.27% (16/220) of patients diagnosed. Disruptions of this gene are recognized to cause cleft (4,23) and palatal anomalies are known to be a core feature of *SATB2*-associated syndrome (24).

Ten percent (23/220) of patients had a P/LP CNV demonstrating the utility of CNV analysis for patients with syndromic OC (Supplementary Material, Table S5). Two single exon duplications were found in *MID1* in three patients (Supplementary Material, Table S5) supporting links between *MID1*-rearrangements and X-linked Opitz G/BBB syndrome (25–27). Microdeletion of chromosome 16p11.2 was also seen in three unrelated patients (1.36%) (Supplementary Material, Table S5) supporting the presence of clefting as a recognized manifestation of this well-characterized recurrent CNV (28). *MAPK3*, which lies within the 16p11.2 recurrent microdeletion region, has been implicated as the cause for the clefting (28). It is, however, important to note that 22q11.2 deletion remains the most common genetic cause of cleft palate and array-CGH is therefore recommended as the first line of testing. Most DDD participants had previous genetic testing which may explain why there is underrepresentation of 22q11.2 deletion patients in this study.

Our study identified 34 genes that now meet criteria to be listed as green in the PA Clefting panel, extending the size of the panel by > 20%. PA applies established gene panels, databases and crowdsourcing to compile phenotype- and disease-specific gene panels (5). In the current study, a reverse phenotypic approach was successfully demonstrated through the review of exome data from a large cohort with a specific phenotype. We suggest that this approach be considered as a means to identify genes for phenotype-specific panels in the future. As panels expand and trait-specific evidence grows, further panels specific to CPO and/or CL/P may be considered.

In addition to the novel candidate genes identified, our patients were found to carry P/LP variants in known causal genes for syndromic (e.g. *EFTUD2*, *PIEZO2*, *CHD7*, *TP63* and *PTCH1*) (15,29) and non-syndromic (e.g. *CDH1*, *TBX22*) (3,13) OC, as well as genes that are known to be important in palate development (e.g. *TBX22*, *BMP2*, *TGFB3*, *SHH*) (3). There were, however, several genes that did not appear in the OxSOC list but may be expected to carry P/LP variants in an unselected cleft cohort: 94 of the green genes in the PA clefting panel did not carry P/LP variants in the OxSOC cohort including *IRF6*, *ARGHAP29*, *FGFR1*, *PAX3* and *MSX1*, all of which are considered to be robust candidate genes for OC (7). Similarly, we report some cases who show an atypical cleft phenotype in relation to existing literature, for example, *KTM2D*, *STAG2* and *KAT6B* (Table 1). These observations demonstrate the rarity of each individual syndrome in the population and illustrate the importance of considering shared phenotypes across syndromes. As we gather more cases, the phenotypic spectrum will become better recognized and described. In part, the absence of robust candidate genes may reflect an ascertainment bias of the DDD study, which focuses upon individuals with unsolved suspected genetic disorders and so effectively removes individuals with exact phenotype to genotype mapping.

The current study aimed to specifically investigate syndromic forms of cleft and to explore genetic heterogeneity across cleft types but this retrospective approach has its limitations. Prospective clinical studies, for example the Cleft Collective (http://www.bristol.ac.uk/dental/cleft-collective/about/), would enable a more detailed genotype–phenotype correlation analysis and a study of larger sample sets, for example the 100 000 Genomes Project, are required for the validation of our findings. Together, such studies will allow us to address questions of heterogeneity within a clinically selected population.

Network analyses highlighted an expected enrichment of broad gene functions related to early embryonic morphogenesis across all subclasses of OC (syndromic, non-syndromic, syndromic CPO and syndromic CL/P, Fig. 3). In particular, epithelial-to-mesenchyme transition, ossification and cell-signalling pathways regulated by *WNT* and *MAPK* were identified. This functional cluster highlights processes and pathways that are known mediators of palate development and reflects findings of network analyses in non-syndromic OC, thereby emphasizing the importance of these processes across all forms of OC (12,13,15,30).

The 34 new green genes identified in the OxSOC cohort forged novel links between the two distinct networks of the existing PA green genes through *ARID1A*, *CHD4*, *SMARCA4*, *SMARCB1* and *TRRAP*, with 8, 10, 10, 7 and 13 connections, respectively (Supplementary Material, Table S8). All of these genes fall within the chromatin organization and/or protein stability functional clusters (Fig. 1) and have previously been associated with neurodevelopmental syndromes. Interestingly, these functions are also known to be relevant to some of the OxSOC genes that were not found to be integrated into our GO networks. For example, SATB2 acts as a docking site for chromatin remodelling enzymes (31), GRHL3 is a pioneer factor that modulates changes in chromatin state (32) and CTNND1 binds the chromatin remodelling protein MORC2 (33). One explanation may be that our GO networks were stringently defined to maximize confidence and included a requirement for experimental evidence of interactions.

More specifically, the five genes that link the PA and OxSOC networks all encode subunits of SWI/SNF (switch/sucrose-non-fermenting) or CHD (chromodomain-helicase-DNA binding) classes of ATP-dependent chromatin remodellers (20), highlighting an additional pathway that has not previously been associated with syndromic OC. SWI/SNF complexes have been described to control *WNT* (34), *SHH* (35) and *MAPK* (36) pathways, indicating that this class may represent higher order regulation of the embryonic morphogenesis cluster found in all subclasses of OC. Chromatin remodelling processes form important master regulators of Neural Crest Cell (NCC) differentiation, maintenance of cell-specific enhancers and neuronal migration and have been associated with a range of neurodevelopmental syndromes (20). Disruption of these processes could therefore have long-lasting and widespread effects on gene expression patterns across many tissues (21,37). This functional cluster therefore has the power to explain the wider phenotype of syndromic OC, either through direct causation or as major contributors within a complex model.

Subsequent gene expression analyses provided further support for the importance of chromatin organization in syndromic OC. Syndromic OC genes in particular showed a higher than average level of expression in early brain tissue (Trimesters 1 and 2) and this was driven by the genes that function in chromatin remodelling and protein stability (Fig. 4). In contrast, non-syndromic OC genes showed a lower than average level of expression in brain and showed inverse patterns of expression in lip and palate tissues when compared with syndromic OC genes. In mandibular/maxillary tissues, a switch in gene expression was observed to occur at time-point E10.5 in both syndromic and non-syndromic gene sets. Previous studies (38) show that the majority of genes involved in normal craniofacial development are upregulated at E9.5 and downregulated at E10.5, coinciding with the patterns observed here for syndromic OC. It is therefore interesting to note that, as a group, non-syndromic OC genes show altered expression at E10.5. This finding may represent altered levels of gene expression or a shift in cell-specific expression patterns and highlights intrinsic differences between syndromic and non-syndromic OC gene expression patterns. Functional studies will be required to uncover the exact cellular mechanisms underlying these differences.

Our paper adds to the current literature in which studies focussing on the genetic architecture of syndromic OC are limited. We apply standardized and accepted guidelines to define pathogenicity in a representative cohort and compare the genes identified with those currently included in clinical panels and in the literature. Nonetheless, some limitations should be noted. As discussed earlier, our study did not discover variants in some genes that are known to be causative of syndromic OC (e.g. *IRF6*). This likely represents the ascertainment of the DDD cohort in which individuals with a genetic diagnosis fully explaining the phenotype were not enrolled. Our conclusions were also limited by the variable phenotypic information available in DECIPHER and a lack of parental segregation testing in some cases. It is likely that the diagnostic rate for the study cohort would have been higher if these limitations were resolved. We applied network and expression analyses to compare genes that contribute to syndromic and non-syndromic forms of OC but differences in the underlying genetic mechanisms make direct comparisons difficult. Syndromic OC is usually monogenic making it relatively simple by comparison to pinpoint a single causative variant, even if functional studies may be needed to demonstrate causality. In comparison, non-familial non-syndromic OCs are multifactorial meaning that gene lists are derived from linkage and association studies and often have little supporting functional evidence. In this study, we chose to work with a non-syndromic gene list derived from a systematic literature review with a manual curation step (CleftDB). Other lists are available and may have given different results but this reflects the field as a whole—there is no gold-standard gene list for either of the OC types investigated here. However, studies such as the one presented here generate a fuller picture of the genetic aetiology and heterogeneity of OC and allow the assessment of candidate genes within this wider picture; a first step in more comprehensive gene lists for future studies.

In conclusion, the molecular pathways associated with syndromic OC have been largely unexplored to date. This report describes a large cohort of patients with syndromic OC and demonstrates a high diagnostic yield from exome sequencing and CNV analysis for patients with syndromic OC. By collating the molecular diagnoses in the studied cohort, we identify 34 genes that now meet criteria to be listed as green in the PA Clefting panel and highlight genes that are commonly disrupted in syndromic OC cases. Our network analyses support previous studies that show that, generally, all forms of cleft (syndromic, non-syndromic, CL/P and CPO) arise from disruption of embryonic morphogenesis. In our analyses, the MAP kinase and WNT cascades were particularly highlighted in both syndromic and non-syndromic forms of OC. Interestingly, our exploratory network and gene expression analyses indicate that the point of disruption differs between syndromic and non-syndromic OC. Syndromic CPO in particular is related to genes involved in ATP-dependent chromatin remodelling. These are highly expressed across both brain and palatal tissue and are critical to the establishment and maintenance of lineage-specific open chromatin regions. Non-syndromic OC is more likely to involve genes that control lip/palate-specific gene expression, perhaps by directing transcription factors to existing regions of open chromatin. These findings support the observation that Genome Wide Association Study (GWAS) significant non-syndromic loci are enriched in enhancer regions (6,13). This study represents a preliminary overview of genetic effects across syndromes in which OC can be a feature. We employed data from one of the few existing cohorts that collected genetic and phenotypic information across developmental syndromes and used existing gene expression datasets to infer information about relative differences in gene expression. The replication of our results will require large cleft-centric cohorts with detailed clinical data to allow direct investigation of mechanisms across OCs. Prospective clinical studies would enable a more detailed genotype–phenotype correlation analysis and larger sample sets (e.g. 100 000 Genomes Project) would enable the investigation of rarer genetic effects in relation to phenotypic outcomes.

## Materials and Methods

Full details of all analyses are available in Supplementary methods, but are presented briefly here.

Six hundred and thirty-one patients included in the DDD study (16,17) with the HPO (https://hpo.jax.org/app/) terms of ‘cleft’ and ‘bifid uvula’ were identified for this Complementary Analysis Project (CAP#5). All patients have undergone exon-array-CGH and exome sequencing (16,17).

All SNVs within known disease-causing genes [in OMIM (https://www.ncbi.nlm.nih.gov/omim/), Clinvar (https://www.ncbi.nlm.nih.gov/clinvar/) or in peer-reviewed medical journals] and CNVs recorded in DECIPHER (https://deciphergenomics.org) (39) for the study cohort were collated. The pathogenicity of SNVs was classified according to the ACMG and the AMP guidelines, the Association for Clinical Genomic Science Best Practice Guidelines and consensus opinion (19). CNVs were classified according to size and gene content in combination with published literature. All genes with P/LP variants were reviewed to determine whether they fulfilled criteria to be classified as ‘green’ in the PA Clefting panel i.e. occurrence in three or more unrelated cases (5).

The function and expression of genes carrying P/LP variants were investigated and compared with a list of non-syndromic candidate genes downloaded from CleftGeneDB (https://bioinfo.uth.edu/CleftGeneDB/). Functional analyses were performed in STRING (40) and GO enrichment analyses in Cytoscape 3.8 (41) using ClueGo (v2.5.8) (42). Average expression data were compared between brain (human) and palatal (mouse and human) tissues in RStudio (43) and included data from Brainspan (https://www.brainspan.org/static/download.html) (44), Gene Expression Omnibus (https://www.ncbi.nlm.nih.gov/geo/) [GSE55965; (38), GDS4921; (45), GDS5071; (46)] and FaceBase (https://www.facebase.org/id/1-SXS2@2X9-ZT1E-1JE2) (47).

## Supplementary Material

Supplementary_Materials_and_Methods_R1_ddad023

References_for_Supplementary_Tables_ddad023

SuppTables_HMG-2022-CE-00520-R1_Wilson_without_ID_ddad023

## Data Availability

The datasets analysed during the current study are available through the DDD (https://www.ddduk.org/access.html). All datasets generated from genetic analyses are provided in the supplementary tables.
