# Supplementary material for: Analysis of exome data in a UK cohort of 603 patients with syndromic orofacial clefting identifies causal molecular pathways
Source: Hum Mol Genet. 2023 Mar 27;32(11):1932–42. doi: 10.1093/hmg/ddad023 (PMC10196673; doi:10.1093/hmg/ddad023)
Supplement: Supplementary_Materials_and_Methods_R1_ddad023 [file supplementary_materials_and_methods_r1_ddad023.docx]

**Supplementary Materials and Methods**

**Cohort**

The DDD study is a national collaborative project in the United Kingdom established to facilitate the translation of genomic sequencing technologies into the National Health Service and to better understand the genetic basis of congenital anomalies and severe undiagnosed developmental disorders (1, 2). In the study 13,612 patients have undergone exon-arrayCGH and exome sequencing. All patients with the Human Phenotype Ontology (HPO) (3) terms of ‘cleft’ and ‘bifid uvula’ were identified within the DDD study through a Complementary Analysis Project (CAP#5). Clefting type was separated into cleft lip +/- palate (CL/P) and CPO (includes and/or bifid uvula). Additional phenotypic data collated included the remaining HPO terms, gender, growth parameters, maternal diabetes during pregnancy, in-utero exposure to anti-epileptic drugs and family history.

**Variant Identification**

‘Plausibly diagnostic’ variants for patients in the study cohort were identified following an automated variant filtering process and manual review (2, 4). The variants were deposited into DECIPHER (DatabasE of genomiC varIation and Phenotype in Humans using Ensembl Resources, <https://deciphergenomics.org>) (5). All single nucleotide variants (SNVs) within known disease-causing genes and copy number variants (CNVs) recorded in DECIPHER for the study cohort were collated. SNVs were classified as pathogenic (P), likely pathogenic (LP), variant of uncertain significance (VUS), likely benign and benign according to the American College of Medical Genetics (ACMG) and the Association for Molecular Pathology (AMP) guidelines, the Association for Clinical Genomic Science (ACGS) Best Practice Guidelines and consensus opinion (4). CNVs were classified according to consensus opinion and published literature.

**PanelApp Analysis**

All genes with P/LP variants (hereon called the ‘Oxford Syndromic Orofacial Clefting (OxSOC) list’) were reviewed to determine whether they are currently included in or fulfilled criteria to be classified as ‘green’ in the PanelApp Clefting panel (6). PanelApp uses a traffic light classification system where green genes are considered to have a high level of evidence to support their use in a clinical setting. Three or more unrelated cases with a variant in the same gene is adequate for that gene to be considered as green (6). Care was taken to avoid double-counting of case numbers as some cases had already been published.

**Gene lists used for network analyses**

A list of genes associated with non-syndromic OC was curated from data available in CleftGeneDB ([https://bioinfo.uth.edu/CleftGeneDB/](https://bioinfo.uth.edu/CleftGeneDB/Download.php?csrt=5798848833676662238)) to facilitate comparative analyses with the genes associated with syndromic OC. CleftGeneDB is a database of 561 experimentally-identified genes associated with cleft lip and/or palate in humans (166 genes) and mice (395 genes) curated by the University of Texas Health Science Centre. References provided by CleftGeneDB were reviewed and genes that were protective factors, maternal risk factors or only associated with syndromic OC were excluded. Genes listed in linkage studies where the studied marker was outside the gene in question were also excluded.

For network analyses, syndromic genes were classified as CPO (N=93) or CL/P (N=77) on the basis of the phenotype seen in OxSOC and current literature. These two groups were mutually exclusive – if a gene met criteria for CL/P, it could not be classified as CPO.

**Network Analyses**

Functional relationships between the genes that carried P/LP variants were investigated within the STRING database (7). A full functional network was built in STRING using experimental, co-expression, neighbourhood and gene fusion sources and a medium confidence level (interaction score >4 ). Functional groups were highlighted as enriched if they had a FDR-value below 0.0001 and an enrichment of at least 1.2-fold above expected.

Gene Ontology (GO) enrichments were performed within Cytoscape 3.8 (8) and relationships between GO terms were investigated using the ClueGo app (v2.5.8) in Cytoscape (9). Clustered functional groups were generated considering all experimental evidence (EXP, IDA, IPI, IMP, IGU, IEP) for GO biological processes, GO cellular components and GO molecular function terms with a p-value ≤0.05. GO terms were fused at a medium network specificity (tree interval between 3 and 8 and a minimum of 3 genes per term and the mapped genes represent at least 4% of the total number of genes in that term with a kappa score of 0.5). A one-sided hypergeometric test (enrichment only) was applied with a Benjamini-Hochberg correction (10).

**Gene Expression Analysis**

OC gene lists were assessed for the presence of differentially expressed genes across one brain and four palatal datasets:

Brain gene expression data were downloaded as normalized RPKM (reads per kilobase per million) values from RNA-seq data from the Brainspan website (Gencode v10 summarised to genes - <https://www.brainspan.org/static/download.html>) (11). This resource includes gene expression data from human brain tissue across the lifespan (between 8pcw and 40 years).

Gene expression in mouse palate was assessed at four time points (E9.5, E10.5, E14.5 and E15.5) using three gene expression resources; (1) an atlas of early mouse craniofacial development (GSE55965) was used to assess gene expression in the emerging mandibular and maxillary arches at E9.5 and E10.5. This dataset included expression array data (Affymetrix Mouse Gene ST I array) downloaded as transformed RMA counts (12), (2) Gene expression in the forming secondary palate was investigated using an RNA-seq dataset from mice at E14.5 (<https://www.facebase.org/id/1-SXS2@2X9-ZT1E-1JE2>). These data were downloaded as transformed TPM normalized expression (13), (3) Gene expression in the fully formed palate was assessed using array data (Affymetrix Mouse Genome 430 2.0 Array) and were downloaded as transformed RMA values (GDS4921) (14).

Gene expression in human palate tissues was approximated using array data (Affymetrix Human Gene 1.0 ST Array) from adult dental pulp samples. This tissue includes mesodermal and neural crest derived cells and therefore share origins with cells involved in lip and palate morphogenesis (GDS5071). These data were downloaded as transformed RMA counts (15).

All normalised datasets underwent the same quality control and analysis steps; genes expressed in <50% of samples were removed from analyses as were transcripts that did not correspond to protein coding genes (as defined by uniprot-proteome_UP000000589 for mice and uniprot-proteome_UP000005640 for humans). Expression levels were winorised at 10% and samples were merged across time-points to generate average expression levels. The final datasets included 16,689 human brain-expressed genes in 119 samples from Trimester 1 (8-13pcw, includes palate formation), 100 from Trimester 2 (14-27pcw), 18 from Trimester 3 (28-40pcw) and 287 postnatal samples (4m-40yr) from different brain regions, 22,325 genes at E9.5 and E10.5 averaged across 11 and 9 wild-type mice respectively, 14,933 genes at E14.5 averaged across 25 wild-type mice, 34,719 genes at E15.5 averaged across 11 wild-type mice and 20,566 human genes in dental pulp averaged across 6 samples from healthy adults. A two-sided t-test was performed in RStudio (16) to compare average expression levels of selected genes lists against all protein coding genes. P-values were then adjusted for the total number of tests performed, using a Benjamini-Hochberg FDR-correction allowing the comparison of relative over-/under-expression of gene sets across tissues (10).

**REFERENCES**

1 Wright, C.F., Fitzgerald, T.W., Jones, W.D., Clayton, S., McRae, J.F., van Kogelenberg, M., King, D.A., Ambridge, K., Barrett, D.M., Bayzetinova, T. *et al.* (2015) Genetic diagnosis of developmental disorders in the DDD study: a scalable analysis of genome-wide research data. *Lancet*, **385**, 1305-1314.

2 Wright, C.F., McRae, J.F., Clayton, S., Gallone, G., Aitken, S., FitzGerald, T.W., Jones, P., Prigmore, E., Rajan, D., Lord, J. *et al.* (2018) Making new genetic diagnoses with old data: iterative reanalysis and reporting from genome-wide data in 1,133 families with developmental disorders. *Genet. Med.*, **20**, 1216-1223.

3 Kohler, S., Gargano, M., Matentzoglu, N., Carmody, L.C., Lewis-Smith, D., Vasilevsky, N.A., Danis, D., Balagura, G., Baynam, G., Brower, A.M. *et al.* (2021) The Human Phenotype Ontology in 2021. *Nucleic Acids Res.*, **49**, D1207-D1217.

4 Richards, S., Aziz, N., Bale, S., Bick, D., Das, S., Gastier-Foster, J., Grody, W.W., Hegde, M., Lyon, E., Spector, E. *et al.* (2015) Standards and guidelines for the interpretation of sequence variants: a joint consensus recommendation of the American College of Medical Genetics and Genomics and the Association for Molecular Pathology. *Genet. Med.*, **17**, 405-424.

5 Firth, H.V., Richards, S.M., Bevan, A.P., Clayton, S., Corpas, M., Rajan, D., Vooren, S.V., Moreau, Y., Pettett, R.M. and Carter, N.P. (2009) DECIPHER: Database of Chromosomal Imbalance and Phenotype in Humans Using Ensembl Resources. *Am. J. Hum. Genet.*, **84**, 524-533.

6 Martin, A.R., Williams, E., Foulger, R.E., Leigh, S., Daugherty, L.C., Niblock, O., Leong, I.U.S., Smith, K.R., Gerasimenko, O., Haraldsdottir, E. *et al.* (2019) PanelApp crowdsources expert knowledge to establish consensus diagnostic gene panels. *Nat. Genet.*, **51**, 1560-1565.

7 Szklarczyk, D., Gable, A.L., Lyon, D., Junge, A., Wyder, S., Huerta-Cepas, J., Simonovic, M., Doncheva, N.T., Morris, J.H., Bork, P. *et al.* (2019) STRING v11: protein-protein association networks with increased coverage, supporting functional discovery in genome-wide experimental datasets. *Nucleic Acids Res.*, **47**, D607-D613.

8 Shannon, P., Markiel, A., Ozier, O., Baliga, N.S., Wang, J.T., Ramage, D., Amin, N., Schwikowski, B. and Ideker, T. (2003) Cytoscape: a software environment for integrated models of biomolecular interaction networks. *Genome Res.*, **13**, 2498-2504.

9 Bindea, G., Mlecnik, B., Hackl, H., Charoentong, P., Tosolini, M., Kirilovsky, A., Fridman, W.H., Pages, F., Trajanoski, Z. and Galon, J. (2009) ClueGO: a Cytoscape plug-in to decipher functionally grouped gene ontology and pathway annotation networks. *Bioinformatics*, **25**, 1091-1093.

10 Benjamini, Y., Heller, R. and Yekutieli, D. (2009) Selective inference in complex research. *Philos Trans A Math Phys Eng Sci*, **367**, 4255-4271.

11 Kang, H.J., Kawasawa, Y.I., Cheng, F., Zhu, Y., Xu, X., Li, M., Sousa, A.M., Pletikos, M., Meyer, K.A., Sedmak, G. *et al.* (2011) Spatio-temporal transcriptome of the human brain. *Nature*, **478**, 483-489.

12 Brunskill, E.W., Potter, A.S., Distasio, A., Dexheimer, P., Plassard, A., Aronow, B.J. and Potter, S.S. (2014) A gene expression atlas of early craniofacial development. *Dev. Biol.*, **391**, 133-146.

13 Grimes, T., Potter, S.S. and Datta, S. (2019) Integrating gene regulatory pathways into differential network analysis of gene expression data. *Sci. Rep.*, **9**, 5479.

14 Iwata, J., Suzuki, A., Yokota, T., Ho, T.V., Pelikan, R., Urata, M., Sanchez-Lara, P.A. and Chai, Y. (2014) TGFbeta regulates epithelial-mesenchymal interactions through WNT signaling activity to control muscle development in the soft palate. *Development*, **141**, 909-917.

15 Kobayashi, G.S., Alvizi, L., Sunaga, D.Y., Francis-West, P., Kuta, A., Almada, B.V., Ferreira, S.G., de Andrade-Lima, L.C., Bueno, D.F., Raposo-Amaral, C.E. *et al.* (2013) Susceptibility to DNA damage as a molecular mechanism for non-syndromic cleft lip and palate. *PLoS One*, **8**, e65677.

16 RStudio Team. (2021) RStudio: Integrated Development Environment for R. First Published on, <http://www.rstudio.com/>.
