# Supplementary material for: Analysis of exome data in a UK cohort of 603 patients with syndromic orofacial clefting identifies causal molecular pathways
Source: Hum Mol Genet. 2023 Mar 27;32(11):1932–42. doi: 10.1093/hmg/ddad023 (PMC10196673; doi:10.1093/hmg/ddad023)
Supplement: References_for_Supplementary_Tables_ddad023 [file references_for_supplementary_tables_ddad023.docx]

**References for Supplementary Tables**

**Table S6**

1. Verloes A, Di Donato N, Masliah-Planchon J, et al. Baraitser–Winter cerebrofrontofacial syndrome: Delineation of the spectrum in 42 cases. Eur J Hum Genet. 2015;23:292-301.
2. Chacon-Camacho OF, Barragan-Arevalo T, Villarroel CE, Almanza-Monterrubio M, Zenteno JC. Previously undescribed phenotypic findings and novel ACTG1 gene pathogenic variants in Baraitser-Winter cerebrofrontofacial syndrome. Eur J Med Genet. 2020;63(5):103877.
3. Lee CG, Jang J, Hyun-Seok J. A novel missense mutation in the ACTG1 gene in a family with congenital autosomal dominant deafness: A case report. Mol Med Rep. 2018;17(6):7611-7617.
4. Mi J, Parthasarathy P, Halliday BJ, et al. Deletion of exon 1 in AMER1 in osteopathia striata with cranial sclerosis. Genes (Basel). 2020;11(12):1439.
5. Hague J, Delon I, Brugger K, et al. Male child with somatic mosaic osteopathia striata with cranial sclerosis caused by a novel pathogenic AMER1 frameshift mutation. Am J Med Genet A. 2017;173(7):1931-1935.
6. Jenkins ZA, van Kogelenberg M, Morgan T, et al. Germline mutations in WTX cause a sclerosing skeletal dysplasia but do not predispose to tumorigenesis. Nat Genet. 2009;41(1):95-100.
7. Goldenberg A, Riccardi F, Tessier A, et al. Clinical and molecular findings in 39 patients with KBG syndrome caused by deletion or mutation of ANKRD11. Am J Med Genet A. 2016;170(11):2847-2859.
8. Low K, Ashraf T, Canham N, et al. Clinical and genetic aspects of KBG syndrome. Am J Med Genet A. 2016;170(11):2835-2846.
9. Van der Sluijs PJ, Jansen S, Vergano SA et al. The ARID1B spectrum in 143 patients: From nonsyndromic intellectual disability to Coffin–Siris syndrome. Genet Med. 2019;21(6):1295-1307.
10. Russell B, Johnston JJ, Biesecker LG, et al. Clinical management of patients with ASXL1 mutations and Bohring-Opitz syndrome, emphasizing the need for Wilms tumor surveillance. Am J Med Genet A. 2015;167A(9):2122-2131.
11. Hoischen A, van Bon BWM, Rodriguez-Santiago B, et al. De novo nonsense mutations of ASXL1 cause Bohring-Opitz syndrome. Nat Genet. 2011;43(8):729-731.
12. Dangiolo SB, Wilson A, Jobanputra V, Anyane-Yeboa K. Bohring–Opitz syndrome (BOS) with a new ASXL1 pathogenic variant: Review of the most prevalent molecular and phenotypic features of the syndrome. Am J Med Genet A. 2015;167A(12):3161-3166.
13. Mosher TM, Zygmunt DA, Koboldt DC, et al. Expansion of B4GALT7 linkeropathy phenotype to include perinatal lethal skeletal dysplasia. Eur J Hum Genet. 2019;27(10):1569-1577.
14. Caraffi SG, Maini I, Ivanovski I, et al. Severe peripheral joint laxity is a distinctive clinical feature of spondylodysplastic-Ehlers-Danlos syndrome (EDS)-B4GALT7 and spondylodysplastic-EDS-B3GALT6. Genes (Basel). 2019;10(10):799.
15. Ragge N, Isidor B, Bitoun P, et al. Expanding the phenotype of the X-linked BCOR microphthalmia syndromes. Hum Genet. 2019;138(8-9):1051-1069.
16. Tan TY, Gonzaga-Jauregui C, Bhoj EJ, et al. Monoallelic BMP2 variants predicted to result in haploinsufficiency cause craniofacial, skeletal, and cardiac features overlapping those of 20p12 deletions. Am J Hum Genet. 2017;101(6):985-994.
17. Ghoumid J, Stichelbout M, Jourdain A-S, et al. Blepharocheilodontic syndrome is a CDH1 pathway–related disorder due to mutations in CDH1 and CTNND1. Genet Med. 2017;19(9):1013-1021.
18. Brioude F, Netchine I, Praz F, et al. Mutations of the imprinted CDKN1C gene as a cause of the overgrowth Beckwith–Wiedemann Syndrome: Clinical spectrum and functional characterization. Hum Mutat. 2015;36(9):894-902.
19. Wakeling EL, Abu Amero S, Alders M, et al. Epigenotype-phenotype correlations in Silver-Russell syndrome. J Med Genet. 2010;47(11);760-768.
20. Weiss K, Lazar HP, Kurolap A, et al. The CHD4-related syndrome: A comprehensive investigation of the clinical spectrum, genotype–phenotype correlations, and molecular basis. Genet Med. 2020;22(2):389-397.
21. van Ravenswaaij-Arts CM, Hefner M, Blake K, Martin D. CHD7 disorder. 2006 [Updated 2020 Sep 17]. In: Adam MP, Ardinger HH, Pagon RA, et al., editors. GeneReviews®_[Internet]. Seattle (WA):University of Washington, Seattle; 1993-2021.
22. Sabbagh S, Antoun S, Megarbane A. CNTNAP1 mutations and their clinical presentations: New case report and systematic review. Case Rep Med. 2020;8795607.
23. Copikova J, Paderova J, Romankova V, et al. Expanding the phenotype spectrum associated with pathogenic variants in the COL2A1 and COL11A1 genes. Ann Hum Genet. 2020;84(5):380-392.
24. Konrad EDH, Nardini N, Caliebe A, et al. CTCF variants in 39 individuals with a variable neurodevelopmental disorder broaden the mutational and clinical spectrum. Genet Med. 2019;21(12):2723-2733.
25. Alharatani R, Ververi A, Beleza-Meireles A, et al. Novel truncating mutations in CTNND1 cause a dominant craniofacial and cardiac syndrome. Hum Mol Genet. 2020;29(11):1900-1921.
26. Snijders Blok L, Madsen E, Juusola J, et al. Mutations in DDX3X are a common cause of unexplained intellectual disability with gender-specific effects on Wnt signaling. Am J Hum Genet. 2015;97(2):343-352.
27. Fieremans N, Van Esch H, Holvoet M, et al. Identification of intellectual disability genes in female patients with a skewed X-inactivation pattern. Hum Mutat. 2016;37(8):804-811.
28. Deciphering Developmental Disorders Study. Large-scale discovery of novel genetic causes of developmental disorders. Nature. 2015;519(7542):223-228.
29. Nowaczyk MJM, Wassif CA. Smith-Lemli-Opitz syndrome. 1998 [Updated 2020 Jan 30]. In: Adam MP, Ardinger HH, Pagon RA, et al., editors. GeneReviews®_[Internet]. Seattle (WA):University of Washington, Seattle; 1993-2021.
30. Bunn KJ, Daniel P, Rosken HS, et al. Mutations in DVL1 cause an osteosclerotic form of Robinow syndrome. Am J Hum Genet. 2015;96(4):623-630.
31. White J, Mazzeu JF, Hoischen A, et al. DVL1 frameshift mutations clustering in the penultimate exon cause autosomal-dominant Robinow syndrome. Am J Hum Genet. 2015:96(4):612-22.
32. Schwartz DD, Fein RH, Carvalho CMB, Sutton VR, Mazzeu JF, Axelrad ME. Neurocognitive, adaptive, and psychosocial functioning in individuals with Robinow syndrome. Am J Med Genet A. 2021;185A:3576-3583.
33. Ullmann U, D’Argenzio L, Mathur S, et al. ECEL1 gene related contractural syndrome: Long-term follow-up and update on clinical and pathological aspects. Neuromuscul Disord. 2018;28(9):741-749.
34. Lines M, Hartley T, MacDonald SK, Boycott KM. Mandibulofacial dysostosis with microcephaly. 2014 [Updated 2020 Nov 12]. In: Adam MP, Ardinger HH, Pagon RA, et al., editors. GeneReviews®_[Internet]. Seattle (WA):University of Washington, Seattle; 1993-2021.
35. Gregor A, Sadleir LG, Asadollahi R, et al. De novo variants in the F-Box Protein FBXO11 in 20 individuals with a variable neurodevelopmental disorder. Am J Hum Genet. 2018;103(2):305-316.
36. Orrico A, Galli L, Cavaliere ML, et al. Phenotypic and molecular characterisation of the Aarskog–Scott syndrome: a survey of the clinical variability in light of FGD1 mutation analysis in 46 patients. Eur J Hum Genet. 2004;12(1):16-23.
37. Volter C, Martinez R, Hagen R, Kress W. Aarskog-Scott syndrome: a novel mutation in the FGD1 gene associated with severe craniofacial dysplasia. Eur J Pediatr. 2014;173(10):1373-1376.
38. Orrico A, Galli L, Obregon MG, de Castro Perez MF, Falciani M, Sorrentino V. Unusually severe expression of craniofacial features in Aarskog-Scott syndrome due to a novel truncating mutation of the FGD1 gene. Am J Med Genet A. 2007;143A(1):58-63.
39. Kobayashi Y, Ogura K, Hikita R, Tsuji M, Moriyama K. Craniofacial, oral, and cervical morphological characteristics in Japanese patients with Apert syndrome or Crouzon syndrome. Eur J Orthod. 2021;43(1):36-44.
40. Slaney SF, Oldridge M, Hurst JA, et al. Differential effects of FGFR2 mutations on syndactyly and cleft palate in Apert syndrome. Am J Hum Genet. 1996;58(5):923-932.
41. Wenger T, Miller D, Evans K. FGFR craniosynostosis syndromes overview. 1998 Oct 20 [Updated 2020 Apr 30]. In: Adam MP, Ardinger HH, Pagon RA, et al., editors. GeneReviews® [Internet]. Seattle (WA): University of Washington, Seattle; 1993-2021.
42. Agochukwu NB, Solomon BD, Doherty ES, Muenke M. The palatal and oral manifestations of Muenke syndrome (FGFR3-related craniosynostosis). J Craniofac Surg. 2012;23(3):664-668.
43. Gonzalez-del Angel A, Caro-Contreras A, Alcantara-Ortigoza MA, Ramos S, Cruz-Alcivar R, Moyers-Perez P. Unique association of hypochondroplasia with craniosynostosis and cleft palate in a Mexican family. Am J Med Genet A. 2018;176(1):161-166.
44. Moutton S, Fergelot P, Naudion S, et al. Otopalatodigital spectrum disorders: refinement of the phenotypic and mutational spectrum. J Hum Genet. 2016;61:693-699.
45. Roberston S. X-linked otopalatodigital spectrum disorders. 2005 Nov 30 [Updated 2019 Oct 3]. In: Adam MP, Ardinger HH, Pagon RA, et al., editors. GeneReviews® [Internet]. Seattle (WA): University of Washington, Seattle; 1993-2021.
46. Bear KA, Solomon BD, Antonini S, et al. Pathogenic mutations in GLI2 cause a specific phenotype that is distinct from holoprosencephaly. J Med Genet. 2014;51(6):413-418.
47. Kaiser FJ, Ansari M, Braunholz D, et al. Loss-of-function HDAC8 mutations cause a phenotypic spectrum of Cornelia de Lange syndrome-like features, ocular hypertelorism, large fontanelle and X-linked inheritance. Hum Mol Genet. 2014;23(11):2888-2900.
48. Au PYB, Goedhard C, Ferguson M, et al. Phenotypic spectrum of Au–Kline syndrome: a report of six new cases and review of the literature. Eur J Hum Genet. 2018;26(9):1272-1281.
49. Zhang LX, Lemire G, Gonzaga-Jauregui C, et al. Further Delineation of the Clinical Spectrum of KAT6B Disorders and Allelic Series of Pathogenic Variants. Genet Med. 2020;22(8):1338-1347.
50. Dunkerton S, Field M, Cho V, et al. A de novo mutation in KMT2A (MLL) in monozygotic twins with Wiedemann–Steiner syndrome. Am J Med Genet A. 2015;167A(9):2182–2187.
51. Li N, Wang Y, Yang Y, et al. Description of the molecular and phenotypic spectrum of Wiedemann-Steiner syndrome in Chinese patients. Orphanet J Rare Dis. 2018;13(1):178.
52. Grangeia A, Leao M, Moura CP. Wiedemann-Steiner syndrome in two patients from Portugal. Am J Med Genet A. 2020;182(1):25-28.
53. Adam MP, Hudgins L, Hannibal M. Kabuki syndrome. 2011 Sep 1 [Updated 2019 Oct 21]. In: Adam MP, Ardinger HH, Pagon RA, et al., editors. GeneReviews® [Internet]. Seattle (WA): University of Washington, Seattle; 1993-2021.
54. Charzewska A, Maiwald R, Kahrizi K, et al. The power of the Mediator complex - Expanding the genetic architecture and phenotypic spectrum of MED12-related disorders. Clin Genet. 2018;94(5):450-456.
55. Caro-Llopis A, Rosello M, Orellana C, et al. De novo mutations in genes of mediator complex causing syndromic intellectual disability: mediatorpathy or transcriptomopathy? Pediatr Res. 2016;80(6):809-815.
56. Rubinato E, Rondeau S, Giuliano F, et al. MED12 missense mutation in a three-generation family. Clinical characterization of MED12-related disorders and literature review. Eur J Med Genet. 2020;63(3);103768.
57. Smol T, Petit F, Piton A, et al. MED13L-related intellectual disability: involvement of missense variants and delineation of the phenotype. Neurogenetics. 2018;19(2):93-103.
58. Gordon CT, Chopra M, Oufadem M, et al. MED13L loss-of-function variants in two patients with syndromic Pierre Robin sequence. Am J Med Genet A. 2018;176(1):181-186.
59. Cafiero C, Marangi G, Orteschi D, et al. Novel de novo heterozygous loss-of-function variants in MED13L and further delineation of the MED13L haploinsufficiency syndrome. Eur J Hum Genet. 2015;23:1499-1504.
60. Li B, Zhou T, Zou Y. Mid1/Mid2 expression in craniofacial development and a literature review of X-linked Opitz syndrome. Mol Genet Genomic Med. 2015;4(1):95-105.
61. Meroni G. X-linked Opitz G/BBB syndrome. 2004 Dec 17 [Updated 2018 Apr 5]. In: Adam MP, Ardinger HH, Pagon RA, et al., editors. GeneReviews® [Internet]. Seattle (WA): University of Washington, Seattle; 1993-2021.
62. Wallgren-Pettersson C, Donner K, Sewry C, et al. Mutations in the nebulin gene can cause severe congenital nemaline myopathy. Neuromuscul Disord. 2002;12(7-8):674-679.
63. Piga D, Magri F, Ronchi D, et al. New mutations in NEB gene discovered by targeted next-generation sequencing in nemaline myopathy Italian patients. J Mol Neurosci. 2016;59(3):351-359.
64. Rocha ML, Dittmayer C, Uruha A, et al. A novel mutation in NEB causing foetal nemaline myopathy with arthrogryposis during early gestation. Neuromuscul Disord. 2021;31(3):239-245.
65. Broix L, Jagline H, Ivanova E, et al. Mutations in the HECT domain of NEDD4L lead to AKT/mTOR pathway deregulation and cause periventricular nodular heterotopia. Nat Genet. 2016;48(11):1349-1358.
66. Toriello HV, Franco B, Bruel AL, Thauvin-Robinet C. Oral-facial-digital syndrome type 1. 2002 Jul 24 [Updated 2016 Aug 4]. In: Adam MP, Ardinger HH, Pagon RA, et al., editors. GeneReviews® [Internet]. Seattle (WA): University of Washington, Seattle; 1993-2021.
67. Abdel-Hamid MS, Issa MY, Otaify GA, Abdel-Ghafar SF, Elbendary HM, Zaki MS. PGAP3-related hyperphosphatasia with mental retardation syndrome: Report of 10 new patients and a homozygous founder mutation.  Clin Genet. 2018;93(1):84-91.
68. Wong SY, Beamer LJ, Gadomski T, et al. Defining the phenotype and assessing severity in phosphoglucomutase-1 deficiency. J Paediatr. 2016;175:130-136.
69. Laumonnier F, Holbert S, Ronce N, et al. Mutations in PHF8 are associated with X linked mental retardation and cleft lip/cleft palate. J Med Genet. 2005;42(10):780-786.
70. McMillin MJ, Beck AE, Chong JX, et al. Mutations in PIEZO2 cause Gordon syndrome, Marden-Walker syndrome, and distal arthrogryposis type 5. Am J Hum Genet. 2014;94(5):734-744.
71. Alisch F, Weichert A, Kalache K, et al. Familial Gordon syndrome associated with a PIEZO2 mutation. Am J Med Genet A. 2017;173(1):254-259.
72. Horn D, Wieczorek D, Metcalfe K, et al. Delineation of PIGV mutation spectrum and associated phenotypes in hyperphosphatasia with mental retardation syndrome. Eur J Hum Genet. 2014;22(6):762-767.
73. Batzir NA, Posey JE, Song X, et al. Phenotypic expansion of POGZ-related intellectual disability syndrome (White-Sutton syndrome). Am J Med Genet A. 2020;182(1):38-52.
74. White J, Beck CR, Harel T, et al. POGZ truncating alleles cause syndromic intellectual disability. Genome Med. 2016;8(1):3.
75. Endo M, Fujii K, Sugita K, Saito K, Kohno Y, Miyashita T. Nationwide survey of nevoid basal cell carcinoma syndrome in Japan revealing the low frequency of basal cell carcinoma. Am J Med Genetic A. 2012;158A(2):351-357.
76. Krab LC, Marcos-Alcalde I, Assaf M, et al. Delineation of phenotypes and genotypes related to cohesion structural protein RAD21. Hum Genet. 2020;139(5):575-592.
77. Niceta M, Barresi S, Pantaleoni F, et al. TARP syndrome: Long-term survival, anatomic patterns of congenital heart defects, differential diagnosis and pathogenic considerations. Eur J Med Genet. 2019;62(6):103534.
78. Zarate YA, Kaylor J, Fish J. SATB2-associated syndrome. 2017 Oct 12. In: Adam MP, Ardinger HH, Pagon RA, et al., editors. GeneReviews® [Internet]. Seattle (WA): University of Washington, Seattle; 1993-2021.
79. Rabin R, Radmanesh A, Glass IA, et al. Genotype-phenotype correlation at codon 1740 of SETD2. Am J Med Genet A. 2020;182(9):2037-2049.
80. Petit F, Escande F, Jourdain AS, et al. Nager syndrome: confirmation of SF3B4 haploinsufficiency as the major cause. Clin Genet. 2014;86(3):246-251.
81. Nanni L, Ming JE, Bocian M, et al. The mutational spectrum of the Sonic Hedgehog gene in holoprosencephaly: SHH mutations cause a significant proportion of autosomal dominant holoprosencephaly. Hum Mol Genet. 1999;8(13):2479-2488.
82. Lacbawan F, Solomon BD, Roessler E, et al. Clinical spectrum of SIX3-associated mutations in holoprosencephaly: correlation between genotype, phenotype and function. J Med Genet. 2009;46(6):389-398.
83. Zechi-Ceide RM, Moura PP, Raskin S, Richieri-Costa A, Guion-Almeida ML. A compound heterozygote SLC26A2 mutation resulting in Robin sequence, mild limbs shortness, accelerated carpal ossification, and multiple epiphyseal dysplasia in two Brazilian sisters. A new intermediate phenotype between diastrophic dysplasia and recessive multiple epiphyseal dysplasia. Am J Med Genet A. 2013;161A(8):2088-2094.
84. Makitie O, Geiberger S, Horemuzova E, et al. SLC26A2 disease spectrum in Sweden – high frequency of recessive multiple epiphyseal dysplasia (rMED). Clin Genet. 2015;87(3):273-278.
85. Kausar M, Makitie RE, Toiviainen-Salo S, Ignatius J, Anees M, Makitie O. Recessive multiple epiphyseal dysplasia – clinical characteristics caused by rare compound heterozygous SLC26A2 genotypes. Eur J Med Genet. 2019;62(11):103573.
86. Kosho T, Okamoto N, Coffin-Siris Syndrome International Collaborators. Genotype-phenotype correlation of Coffin-Siris syndrome caused by mutations in SMARCB1, SMARCA4, SMARCE1, and ARID1A. Am J Med Genet C Semin Med Genet. 2014;166C(3):262-275.
87. Huisman S, Mulder PA, Redeker E, et al. Phenotypes and genotypes in individuals with SMC1A variants. Am J Med Genet A. 2017;173(8):2108-2125.
88. Mattos EP, Sanseverino MTV, Magalhaes JAA, et al. Clinical and molecular characterization of a Brazilian cohort of campomelic dysplasia patients, and identification of seven new SOX9 mutations. Genet Mol Biol. 2015;38(1):14-20.
89. Bhoj EJ, Haye D, Toutain A, et al. Phenotypic spectrum associated with SPECC1L pathogenic variants: new families and critical review of the nosology of Teebi, Opitz GBBB, and Baraitser-Winter syndromes. Eur J Med Genet. 2019;62(12):103588.
90. Aoi H, Lei M, Mizuguchi T, et al. Nonsense variants of STAG2 result in distinct congenital anomalies. Hum Genome Var. 2020;7:26.
91. Soardi FC, Machado-Silva A, Linhares ND, et al. Familial STAG2 germline mutation defines a new human cohesinopathy. NPJ Genom Med. 2017;2:7.
92. Marcano ACB, Doudney K, Braybrook C, et al. TBX22 mutations are a frequent cause of cleft palate. J Med Genet. 2004;41(1):68-74.
93. Vincent M, Genevieve D, Ostertag A, et al. Treacher Collins syndrome: a clinical and molecular study based on a large series of patients. Genet Med. 2016;18(1):49-56.
94. Teber OA, Gillessen-Kaesbach G, Fischer S, et al. Genotyping in 46 patients with tentative diagnosis of Treacher Collins syndrome revealed unexpected phenotypic variation. Eur J Hum Genet. 2004;12(11):879-890.
95. Lin AE, Haldeman-Englert CR, Milunsky JM. Branchiooculofacial syndrome. 2011 May 31 [Updated 2018 Mar 29]. In: Adam MP, Ardinger HH, Pagon RA, et al., editors. GeneReviews® [Internet]. Seattle (WA): University of Washington, Seattle; 1993-2021.
96. Miller DE, Chow P, Gallagher ER, Perkins JA, Wenger TL. Catel-Manzke syndrome without Manzke dysostosis. Am J Med Genet A. 2020;182(3):437-440.
97. Loeys BL, Dietz HC. Loeys-Dietz syndrome. 2008 Feb 28 [Updated 2018 Mar 1]. In: Adam MP, Ardinger HH, Pagon RA, et al., editors. GeneReviews® [Internet]. Seattle (WA): University of Washington, Seattle; 1993-2021.
98. Sharkia R, Zalan A, Jabareen-Masri A, et al. A novel biallelic loss-of-function mutation in TMCO1 gene confirming and expanding the phenotype spectrum of cerebro-facio-thoracic dysplasia. Am J Med Genet A. 2019;179(7)1338-1345.
99. Sutton VR, van Bokhoven H. TP63-related disorders. 2010 Jun 8 [Updated 2019 Dec 5]. In: Adam MP, Ardinger HH, Pagon RA, et al., editors. GeneReviews® [Internet]. Seattle (WA): University of Washington, Seattle; 1993-2020.
100. Castilla-Vallmanya L, Selmer KK, Dimartino C, et al. Phenotypic spectrum and transcriptomic profile associated with germline variants in TRAF7. Genet Med. 2020;22(7):1215-1226.
101. Cogne B, Ehresmann S, Beauregard-Lacroix E, et al. Missense variants in the histone acetyltransferase complex component gene TRRAP cause autism and syndromic intellectual disability. Am J Hum Genet. 2019;104(3):530-541.
102. Reijnders MRF, Zachariadis V, Latour B, et al. De novo loss-of-function mutations in USP9X cause a female-specific recognizable syndrome with developmental delay and congenital malformations. Am J Hum Genet. 2016;98(2):373-81.
103. Frints SGM, Hennig F, Colombo R, et al. Deleterious de novo variants of X-linked ZC4H2 in females cause a variable phenotype with neurogenic arthrogryposis multiplex congenita. Hum Mutat. 2019;40(12):2270-2285.
104. Van Dijck A, Vulto-van Silfhout AT, Cappuyns E, et al. Clinical presentation of a complex neurodevelopmental disorder caused by mutations in ADNP. Biol Psychiatry. 2019;85(4):287-297.
105. Saeki S, Enokizono T, Imagawa K, et al. A case of autism spectrum disorder with cleft lip and palate carrying a mutation in exon 8 of AUTS2. Clin Case Rep. 2019;7(11):2059-2063.
106. Uehara T, Takenouchi T, Kosaki R, Kurosawa K, Mizuno S, Kosaki K. Redefining the phenotypic spectrum of de novo heterozygous CDK13 variants: Three patients without cardiac defects. Eur J Med Genet. 2018;61(5):243-247.
107. Drivas TG, Li D, Nair D, et al. A second cohort of CHD3 patients expands the molecular mechanisms known to cause Snijders Blok-Campeau syndrome. Eur J Hum Genet. 2020;28(10):1422-1431.
108. Fergelot P, Van Belzen M, Van Gils J, et al. Phenotype and genotype in 52 patients with Rubenstein-Taybi syndrome caused by EP300. Am J Med Genet A. 2016;170(12):3069-3082.
109. Kennedy J, Goudie D, Blair E, et al. KAT6A syndrome: Genotype-phenotype correlation in 76 patients with pathogenic KAT6A variants. Genet Med. 2019;21(4):850-860.
110. Koolen DA, Pfundt R, Linda K, et al. The Koolen-de Vries syndrome: a phenotypic comparison of patients with a 17q21.31 microdeletion versus a KANSL1 sequence variant. Eur J Hum Genet. 2016;24(5):652-659.
111. Patak J, Gilfert J, Byler M, et al. MAGEL2-related disorders: A study and case series. Clin Genet. 2019;96(6):493-505.
112. Gregory LC, Shah P, Sanner JRF, et al. Mutations in MAGEL2 and L1CAM are associated with congenital hypopituitarism and arthrogryposis. J Clin Endocrinol Metab. 2019;104(12):5737-5759.
113. Mircsof D, Langouet M, Rio M, et al. Mutations in NONO lead to syndromic intellectual disability and inhibitory synaptic defects. Nat Neurosci. 2015;18(12):1731-1736.
114. Midro AT, Stasiewicz-Jarocka B, Borys J, et al. A 23-year follow-up of a male with Hajdu-Cheney syndrome due to NOTCH2 mutation. Am J Med Genet A. 2018;176(11):2382-2388.
115. Crifasi PA, Patterson MC, Bonde D, Michels VV. Severe Hajdu-Cheney syndrome with upper airway obstruction. Am J Med Genet. 1997;70(3):261-266.
116. Regev M, Pode-Shakked B, Jacobson JM, Raas-Rothschild A, Goldstein DB, Anikster Y. Phenotypic variability in Hajdu-Cheney syndrome. Eur J Med Genet. 2019;62(1):35-38.
117. Van Esch H, Colnaghi R, Freson K, et al. Defective DNA polymerase α-primase leads to X-linked intellectual disability associated with severe growth retardation, microcephaly, and hypogonadism. Am J Hum Genet. 2019;104(5):957-967.
118. Haijes HA, Koster MJE, Rehmann H, et al. De novo heterozygous POLR2A variants cause a neurodevelopmental syndrome with profound infantile-onset hypotonia. Am J Hum Genet. 2019;105(2):283-301.
119. El Chehadeh S, Kerstjens-Frederikse WS, Thevenon J, et al. Dominant variants in the splicing factor PUF60 cause a recognizable syndrome with intellectual disability, heart defects and short stature. Eur J Hum Genet. 2017;25(1):43-51.
120. Low KJ, Ansari M, Jamra RA, et al. PUF60 variants cause a syndrome of ID, short stature, microcephaly, coloboma, craniofacial, cardiac, renal and spinal features. Eur J Hum Genet. 2017;25(1):552-559.
121. Fregeau B, Kim BJ, Hernandez-Garcia A, et al. De novo mutations of RERE cause a genetic syndrome with features that overlap those associated with proximal 1p36 deletions. Am J Hum Genet. 2016;98(5):963-970.
122. Balasubramanian M, Dingemans AJM, Albaba S, et al. Comprehensive study of 28 individuals with SIN3A-related disorder underscoring the associated mild cognitive and distinctive facial phenotype. Eur J Hum Genet. 2021;29(4):625-636.
123. Kruszka P, Berger SI, Vasa C, et al. Cohesin complex-associated holoprosencephaly. Brain. 2019;142(9):2631-2643.
124. Khan U, DDD Study, Baker E, Clayton-Smith J. Observation of cleft palate in an individual with SOX11 mutation: Indication of a role for SOX11 in human palatogenesis. Cleft Palate Craniofac J. 2018;55(3):456-461.
125. Slavotinek A, Pua H, Hodoglugil U, et al. Pierpont syndrome associated with the p.Tyr446Cys missense mutation in TBL1XR1. Eur J Med Genet. 2017;60(10):504-508.
126. Sharma V, Fenwick AL, Brockop MS, et al. Mutations of TCF12, encoding a basic-helix-loop-helix partner of TWIST1, are a frequent cause of coronal craniosynostosis. Nat Genet. 2013;45(3):304-307.
127. Yilmaz E, Mihci E, Nur B, Alper OM. Coronal craniosynostosis due to TCF12 mutations in patients from Turkey. Am J Med Genet A. 2019;179(11):2241-2245.
128. Flex E, Ciolfi A, Caputo V, et al. Loss of function of the E3 ubiquitin-protein ligase UBE3B causes Kaufman oculocerebrofacial syndrome. J Med Genet. 2013;50(8):493-499.
129. Olley G, Ansari M, Bengani H, et al. BRD4 interacts with NIPBL and BRD4 is mutated in a Cornelia de Lange-like syndrome. Nat Genet. 2018;50(3):329-332.
130. Banka S, Sayer R, Breen C, et al. Genotype-phenotype specificity in Menke-Hennekam syndrome caused by missense variants in exon 30 or 31 of CREBBP. Am J Med Genet A. 2019;179(6):1058-1062.
131. van Bon BWM, Coe BP, de Vries BBA, Eichler EE. DYRK1A-related intellectual disability syndrome. 2015 Dec 17. In: Adam MP, Ardinger HH, Pagon RA, et al., editors. GeneReviews® [Internet]. Seattle (WA): University of Washington, Seattle; 1993-2020.
132. Martin HC, Jones WD, McIntyre R, et al. Quantifying the contribution of recessive coding variation to developmental disorders. Science. 2018;362(6419):1161-1164.
133. Durkin A, Albaba S, Fry AE, et al. Clinical findings of 21 previously unreported probands with HNRNPU-related syndrome and comprehensive literature review. Am J Med Genet A. 2020;182(7):1637-1654.
134. Chrzanowska K, Fryns J-P, Van den Berghe H. Cardio-facio-cutaneous (CFC) syndrome: Report of a new patient. Am J Med Genet. 1989;33(4):471-473.
135. Skraban CM, Wells CF, Markose P, et al. WDR26 haploinsufficiency causes a recognizable syndrome of intellectual disability, seizures, abnormal gait, and distinctive facial features. Am J Hum Genet. 2017;101(1):139-148.
